# Proposals for Improving Maternal Safety (2024 Edition): Insights From the Analysis of Maternal Deaths in Japan

**DOI:** 10.1111/jog.70302

**Published:** 2026-05-12

**Authors:** Japan Maternal Death Exploratory Committee, Murasaki Aman, Tatsuya Arakaki, Masayuki Endo, Junichi Hasegawa, Koji Hashii, Masako Hayashi, Yuki Hosokawa, Tomoaki Ikeda, Hiroshi Ishikawa, Isamu Ishiwata, Chizuko A. Kamiya, Takeshi Kanagawa, Naohiro Kanayama, Rie Kato, Shinji Katsuragi, Takao Kobayashi, Tomomi Kotani, Takahiko Kubo, Kentaro Kurasawa, Yoshiki Maeda, Takahide Maenaka, Shintaro Makino, Hiroshi Matsumoto, Kiyonori Miura, Takeshi Murakoshi, Akihito Nakai, Masamitsu Nakamura, Masahiko Nakata, Masafumi Nii, Keiji Nogami, Tomoaki Oda, Toshiyuki Okutomi, Kazuhiro Osato, Yoko Sagara, Atsushi Sakurai, Shoji Sato, Akihiko Sekizawa, Yumi Shina, Hiroyuki Sumikura, Toshihito Suzuki, Jun C Takahashi, Mayumi Takano, Satoru Takeda, Noboru Tanabe, Kayo Tanaka, Hiroaki Tanaka, Katsuo Terui, Nobuya Unno, Tomoko Wakasa, Tomoyuki Yamashita, Kazuma Yamakawa, Takaaki Yasuda, Jun Yoshimatsu

**Affiliations:** ^1^ Department of Obstetrics and Gynecology Saiseikai Matsusaka General Hospital Matsusaka Mie Japan

**Keywords:** hyperemesis gravidarum, maternal death, Mississippi protocol, obstetric hemorrhage, suicide

## Abstract

The Proposals for Improving Maternal Safety (2024 Edition) were developed by the Japan Maternal Death Exploratory Committee and the Japan Association of Obstetricians and Gynecologists based on systematic reviews of 640 maternal deaths reported nationwide between 2010 and 2024. Obstetric hemorrhage remained the leading cause of maternal death, followed by intracranial hemorrhage, amniotic fluid embolism, suicide, cardiovascular disease, infection, and pulmonary disease. In 2024, maternal deaths increased sharply to 47, largely driven by a resurgence of hemorrhage‐related deaths, while suicide has persistently ranked among the top causes in recent years. Detailed analysis revealed preventable system‐level failures in diagnosis, timing of intervention, and interdisciplinary collaboration. Based on these findings, five key proposals are presented: (1) Immediate recognition and correction of consumptive coagulopathy in placental abruption with fetal death, emphasizing early fibrinogen measurement and replacement. (2) Prompt re‐laparotomy when cesarean suture dehiscence is suspected, as balloon tamponade and arterial embolization are ineffective for structural lesions. (3) Active implementation of the Mississippi protocol for HELLP (hemolysis, elevated liver enzyme levels, and low platelet count) syndrome to prevent fatal cerebral hemorrhage. (4) Prevention of maternal suicide through regional collaboration among obstetrics, psychiatry, and administrative agencies, combined with a population‐based mental health approach. (5) Appropriate and proactive intervention for hyperemesis gravidarum, recognizing its association with severe physical complications and maternal mental health deterioration. These proposals highlight urgent priorities for clinical practice, education, and system reform to reduce preventable maternal deaths in Japan.

Proposals for Improving Maternal Safety (2024 Edition), created by the Japan Maternal Death Exploratory Committee (JMDEC) and the Japan Association of Obstetricians and Gynecologists (JAOG), was released in October 2025 [1]. An English‐language version of these proposals was created based on the contents of the original Japanese‐language version.

## 1 Maternal Death Report System

Since its establishment in 2010, JMDEC and JAOG have continuously collected and analyzed detailed reports of maternal deaths submitted nationwide. Each case is reviewed by a multidisciplinary panel, including obstetricians, anesthesiologists, emergency physicians, psychiatrists, and other specialists, to determine the most probable cause of death and identify preventable factors.

### 1.1 The Classification of Causes of Death and Recent Trend

Although ICD‐based classification has advantages—such as identifying background conditions like hypertensive disorders of pregnancy—disease‐based categorization remains intuitive and clinically meaningful; 640 maternal deaths which occurred during 15 years from 2010 to 2024, obstetric hemorrhage (18%) was the leading cause of maternal death, followed by intracranial hemorrhage or infarction (14%), cardiopulmonary collapse–type amniotic fluid embolism (11%), suicide (11%), cardiovascular disease (9%), infection (8%), and pulmonary diseases including pulmonary embolism (8%).

**FIGURE 1:**
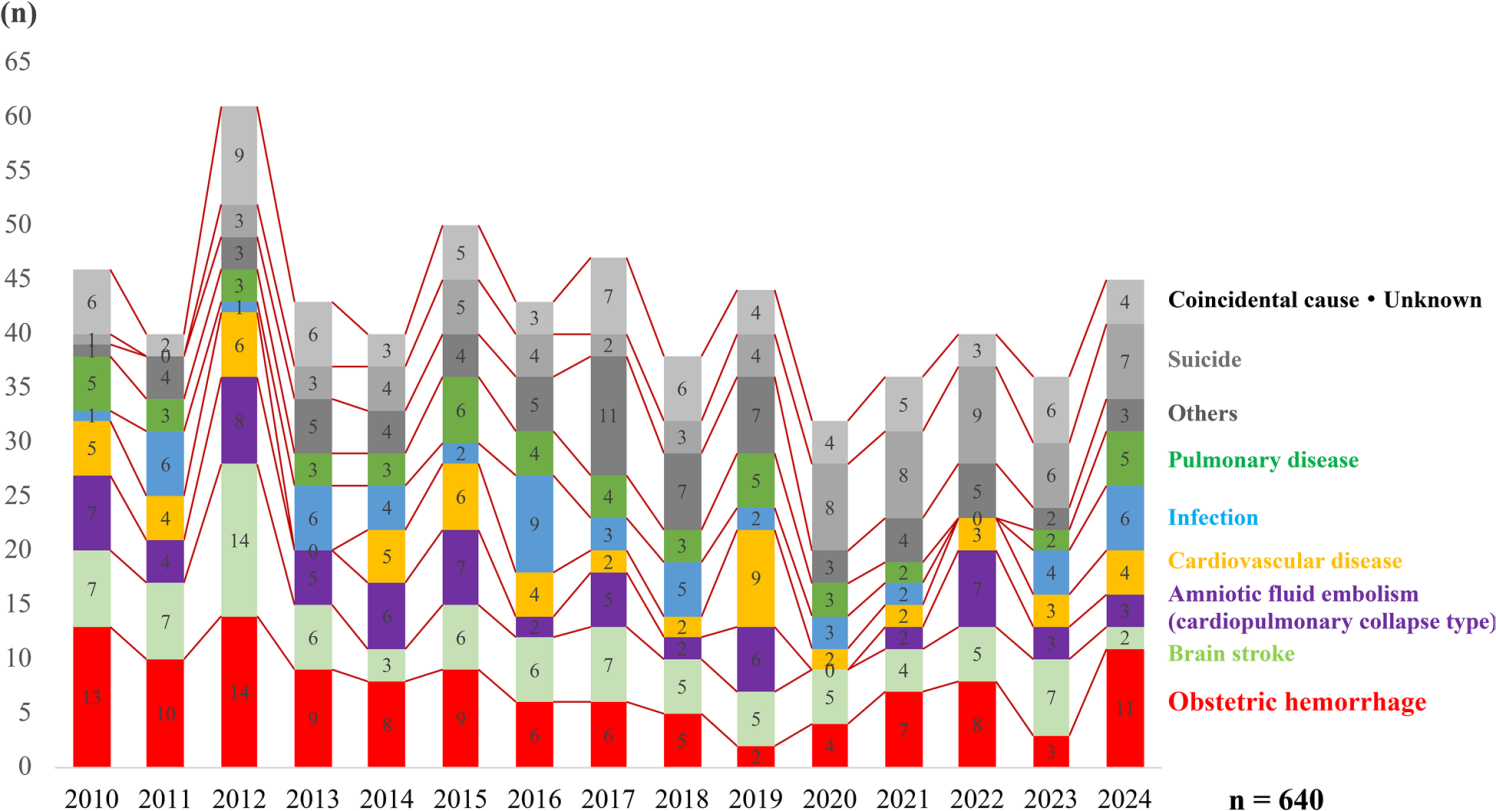
Annual trends of the cause of maternal death.

In 2024, the number of maternal deaths increased sharply to 47, largely driven by obstetric hemorrhage. The proportion of deaths attributable to obstetric hemorrhage declined markedly from 13 cases (28% of all maternal deaths) in 2010 to 2 cases (5%) in 2019. However, from 2020 onward, hemorrhage‐related deaths increased again, reaching 11 cases (24%) in 2024—levels comparable to those observed at the initiation of the project. In contrast, over the past 5 years, suicide has consistently remained among the leading causes of maternal death, whereas cardiopulmonary collapse–type amniotic fluid embolism, cardiovascular disease, infection, and pulmonary disease have shown relatively stable trends (Figure 1).

**FIGURE 2:**
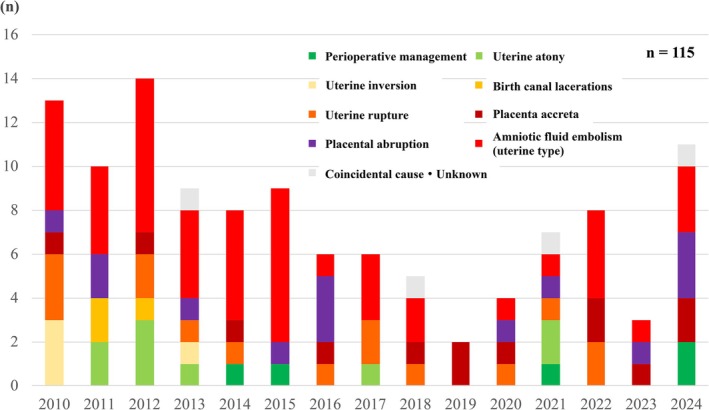
Annual trends in the causes of obstetric hemorrhage.

The annual trends in the causes of obstetric hemorrhage are shown in Figure 2. Causes not primarily related to coagulation disorders are depicted in yellow and green tones, whereas coagulation‐related causes are shown in red and purple tones. At the outset of this project, commonly encountered causes, such as uterine atony and birth canal lacerations, occurred as frequently as sudden‐onset causes, including uterine‐type amniotic fluid embolism and placental abruption. Following the dissemination of recommendations for maternal safety and the establishment of Japan Council for Implementation of Maternal Emergency Life‐Saving System, perinatal management improved, leading to a progressive decline in deaths from obstetric hemorrhage due to common causes. In contrast, recent increases in hemorrhage‐related deaths have been driven predominantly by placental abruption, placenta accreta, and hemorrhagic complications associated with cesarean section. The increase in placenta accreta is likely related to advances in reproductive medicine and rising cesarean section rates. These findings underscore the need for renewed and focused education on the management of obstetric hemorrhage.

## 2 Proposal 1

Immediate recognition and correction of consumptive coagulopathy in placental abruption with fetal death, emphasizing early fibrinogen measurement and replacement.

### 2.1 Case Summary

A multiparous woman in her 30s with a previous cesarean section presented at 37 weeks' gestation with sudden severe lower abdominal pain. She had a board‐like abdomen and intrauterine fetal death. Emergency cesarean section confirmed placental abruption with marked Couvelaire uterus and massive hemorrhage. Despite surgical intervention, blood loss exceeded 3000 g, necessitating maternal transfer. On arrival, she was in profound shock with < 50 mg/dL of blood fibrinogen level and cardiac arrest occurred within 30 min despite intensive resuscitation.

### 2.2 Comment

Placental abruption complicated by fetal death usually indicates a prolonged or extensive separation, leading to severe consumption coagulopathy. Data from the Japan Society of Obstetrics and Gynecology Perinatal Database (2020–2022) revealed 5965 cases of abruption, of which 411 (6.9%) involved intrauterine fetal death. Compared to cases without fetal death, these had significantly higher risks of hemorrhage > 2000 mL (9.4‐fold), obstetric disseminated intravascular coagulation (DIC; 11.1‐fold), and transfusion (7.5‐fold) (unpublished data).

In many primary hospitals, coagulation tests are delayed or unavailable. This diagnostic lag prevents the referral hospital from preparing adequate blood products. Fibrinogen measurement is the most critical single parameter—values < 150 mg/dL are strongly predictive of DIC progression. After the 2023 insurance approval for fibrinogen concentrate, requests for point‐of‐care testing (POCT) devices have increased [2], reflecting clinical demand for rapid evaluation. When placental abruption with fetal death is diagnosed, blood sampling for fibrinogen and coagulation factors must be done immediately with intravenous line placement, and results should be shared with the referral center. Early transfusion of fibrinogen concentrate and fresh frozen plasma is life‐saving. Many fatal cases reveal two patterns of error: (1) coagulation not measured at diagnosis, and (2) coagulopathy recognized but replacement therapy delayed. Both reflect system‐level gaps that can be resolved by standardized POCT‐based pathways.

In cases of placental abruption with fetal death, fibrinogen and coagulation parameters should be measured immediately. If hypofibrinogenemia is detected, coagulation factor replacement must be initiated without delay.

## 3 Proposal 2

Prompt re‐laparotomy when cesarean suture dehiscence is suspected, as balloon tamponade and arterial embolization are ineffective for structural lesions.

### 3.1 Case Summary

A primiparous woman in her 40s with multiple fibroids underwent emergency cesarean section for fetal distress. Postoperatively, massive bleeding persisted despite balloon tamponade and uterine artery embolization. She died of hemorrhagic shock; autopsy revealed uterine incision dehiscence and uterine artery rupture.

### 3.2 Comment

Post‐cesarean hemorrhage may result from suture dehiscence or unrecognized extension of the incision, particularly when the lower uterine segment is thin, effaced, or myomatous [3]. In such circumstances, low transverse incisions under poor visualization can easily extend laterally, injuring the uterine artery. When postoperative bleeding exceeds expectation, physicians often attempt balloon tamponade or embolization. However, these methods do not address structural disruption and may worsen bleeding by delaying definitive repair. Uterine artery embolization is effective for atonic bleeding but ineffective for mechanical lesions such as uterine rupture, incision dehiscence, or placenta accreta.

**FIGURE 3:**
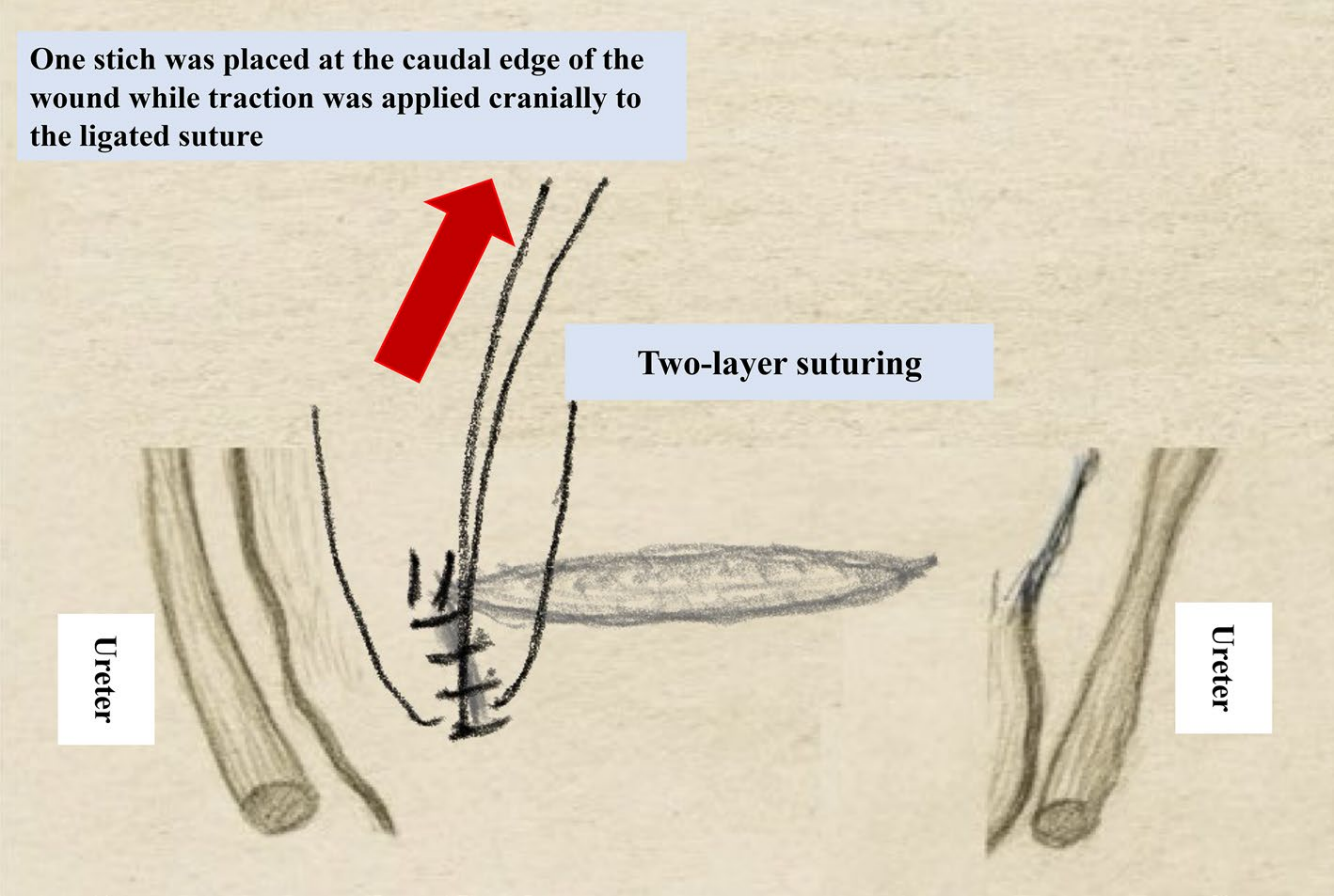
Pull‐and‐Suture method.

The key principle is “open, confirm, and repair”. Once suture failure is suspected, immediate re‐laparotomy should be undertaken. Direct visualization permits targeted hemostasis using techniques such as the modified O'Leary stitch—suturing the uterine artery and surrounding myometrium 2–3 cm above and below the incision, while retracting the uterus cranially and contralaterally to protect the ureter [4]. When cervical or lower‐segment tears extend downward, the Pull‐and‐Suture method (Figure 3) allows controlled approximation of deep lacerations.

**FIGURE 4:**
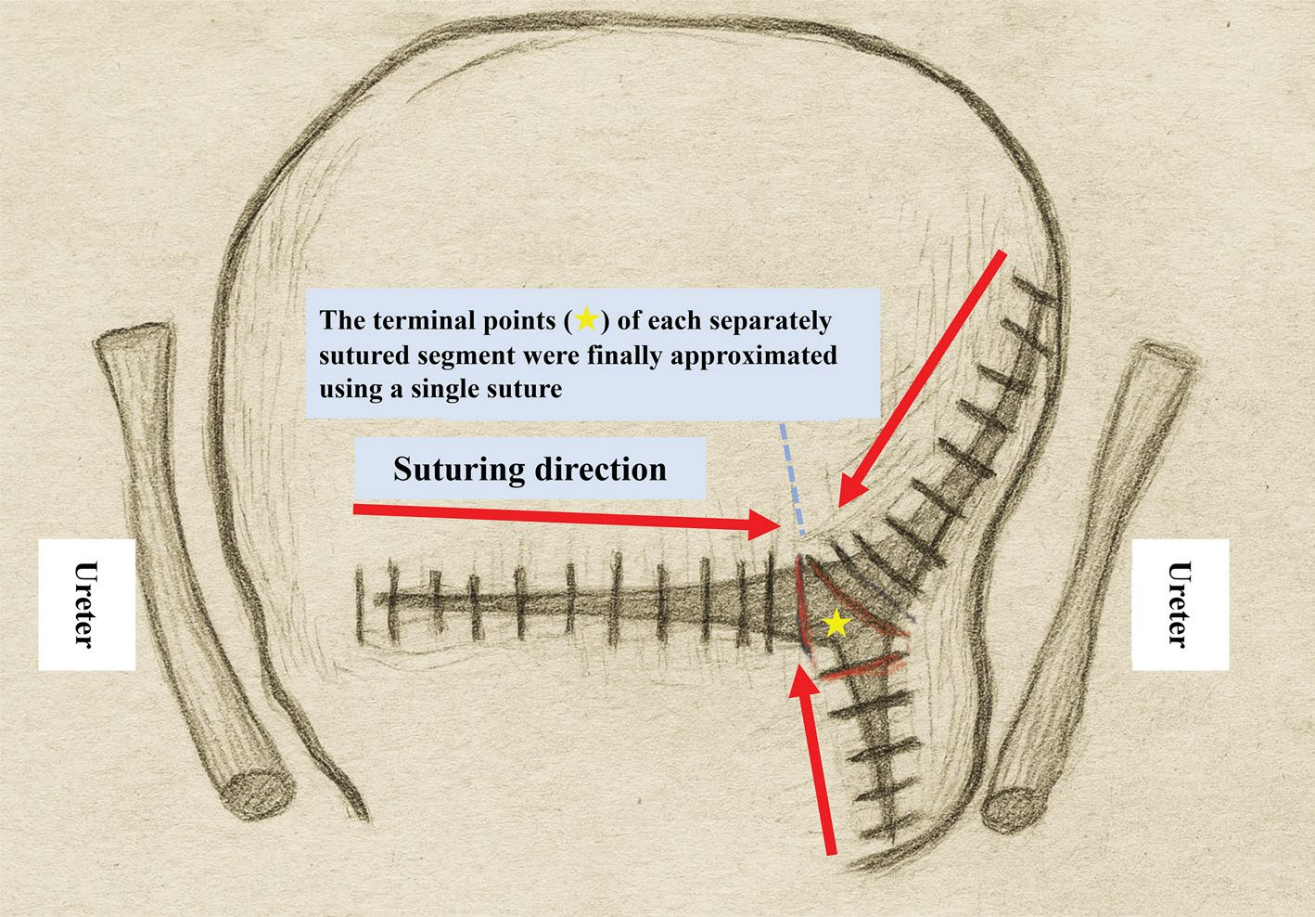
Multiple‐area integrated suturing (MAIS).

Where multiple complex laceration edges are present, suture each section separately (multiple‐area integrated suturing (MAIS), Figure 4). During the multiple‐point suturing approach, the Pull and Suture method may be employed to ensure secure suturing of the laceration edges.

Many fatal cases stemmed from hesitation to reopen the abdomen or overreliance on interventional radiology. Obstetric teams must recognize that timely surgical re‐entry saves lives, even if minimally invasive options appear tempting.

## 4 Proposal 3

Active implementation of the Mississippi protocol for HELLP (hemolysis, elevated liver enzyme levels, and low platelet count) syndrome to prevent fatal cerebral hemorrhage.

### 4.1 Case Summary

A primiparous woman in her 30s had antenatal care at a clinic. From 22 weeks, she showed hypertension and proteinuria. At 30 weeks, she reported epigastric pain and was advised to visit a general hospital. Despite severe hypertension and abnormal labs suggestive of HELLP syndrome, she was diagnosed with gastroenteritis and discharged. Hours later, she collapsed at home. Intracerebral hemorrhage was identified. Despite emergency neurosurgery and cesarean delivery, she died.

### 4.2 Comment

HELLP syndrome often evolves rapidly from preeclampsia and may precede laboratory confirmation. The patient's fatal outcome reflects a common pitfall—misinterpretation by non‐obstetric physicians and underestimation of early warning signs such as upper abdominal pain.

Japan's maternal death investigations reveal a distinct pattern: hemorrhagic stroke accounts for 70%–75% of cases, in contrast to the predominance of ischemic stroke in Western countries. Genetic predisposition to vascular fragility and heightened sensitivity to hypertension have been suggested as contributing factors. Given that hemorrhagic stroke is associated with more severe and often fatal outcomes than ischemic stroke, intensive preventive management tailored to Japan's cerebrovascular risk profile is essential. The Mississippi protocol, which integrates magnesium sulfate, aggressive blood pressure control, and corticosteroid therapy, remains the most effective structured approach [5].

- Magnesium sulfate: A 4‐g intravenous loading dose is administered over ≥ 20 min, followed by a continuous infusion of 1 g/h for ≥ 24 h postpartum, with serum magnesium levels maintained at 4–8 mEq/L.
- Blood pressure control: When systolic blood pressure is ≥ 160 mmHg, it is reduced to < 140 mmHg using a continuous nicardipine infusion.
- Corticosteroids: Dexamethasone 10 mg is administered intravenously every 12 h until the platelet count exceeds 100 × 10^9^/L.

Martin et al. reported zero maternal deaths among 190 Class I–II cases under this regimen [6]. In Japan's 2020–2022 perinatal database (*n* = 1461 HELLP cases), 22.7% received corticosteroids; no cerebral hemorrhage or maternal death occurred in this group, while four each occurred among non‐treated cases (unpublished data). This finding reinforces the protocol's protective role.

Despite this, the Mississippi protocol remains underutilized in Japan, partly due to concerns about steroid efficacy and limited awareness among general physicians. Yet, active application—especially of magnesium and antihypertensive control—can markedly reduce fatal intracranial bleeding.

## 5 Proposal 4

Prevention of maternal suicide through regional collaboration among obstetrics, psychiatry, and administrative agencies, combined with a population‐based mental health approach.

**FIGURE 5:**
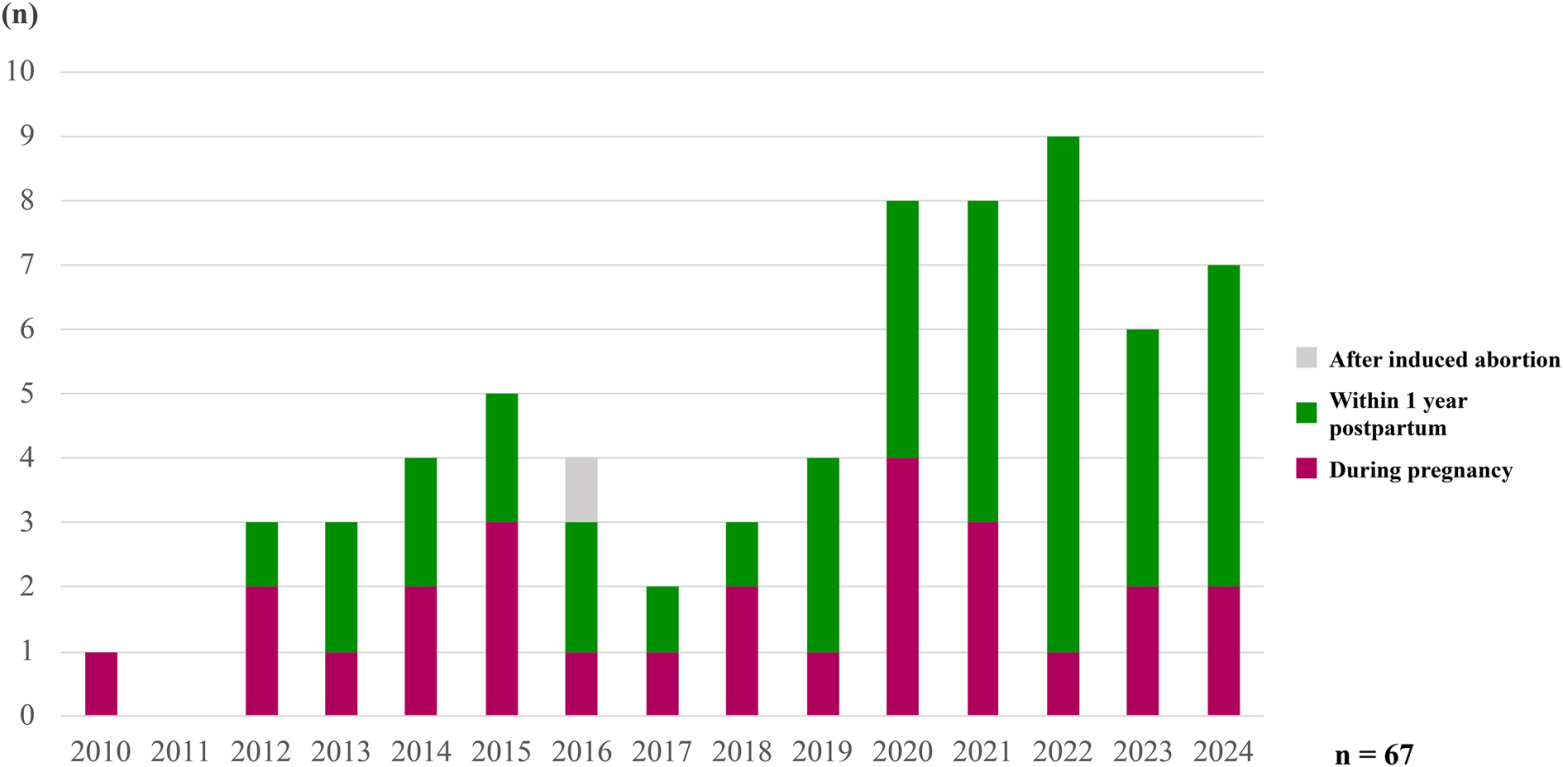
Annual trends in maternal deaths due to suicide.

Maternal suicide has become an increasingly important cause of maternal death in Japan, particularly during the postpartum period. As shown in Figure 5, the proportion of maternal deaths attributed to suicide has risen in recent years. This trend partly reflects a change in recognition, whereby the psychological impact of pregnancy and childbirth is now considered relevant even in late maternal deaths that were previously overlooked. Furthermore, revision of the national suicide surveillance system in 2022 to include pregnancy and postpartum status revealed substantially higher numbers of maternal suicides than those identified through conventional maternal death reviews, indicating that suicide prevention is a critical component of maternal safety.

### 5.1 Case Summary

A primiparous woman in her 30s had a history of depression and emotionally unstable personality disorder, with prior suicide attempts, but was stable and cleared for pregnancy. She received antenatal care at a general hospital with psychiatric support. Although obstetric progress was uneventful, she developed insomnia, physical distress, and suicidal thoughts during mid‐to‐late pregnancy. Despite an emergency psychiatric visit without admission, she was found hanging at home 3 days later. Family interviews revealed worsening nausea, insomnia, and anxiety about childbirth and parenting.

### 5.2 Comment

**FIGURE 6:**
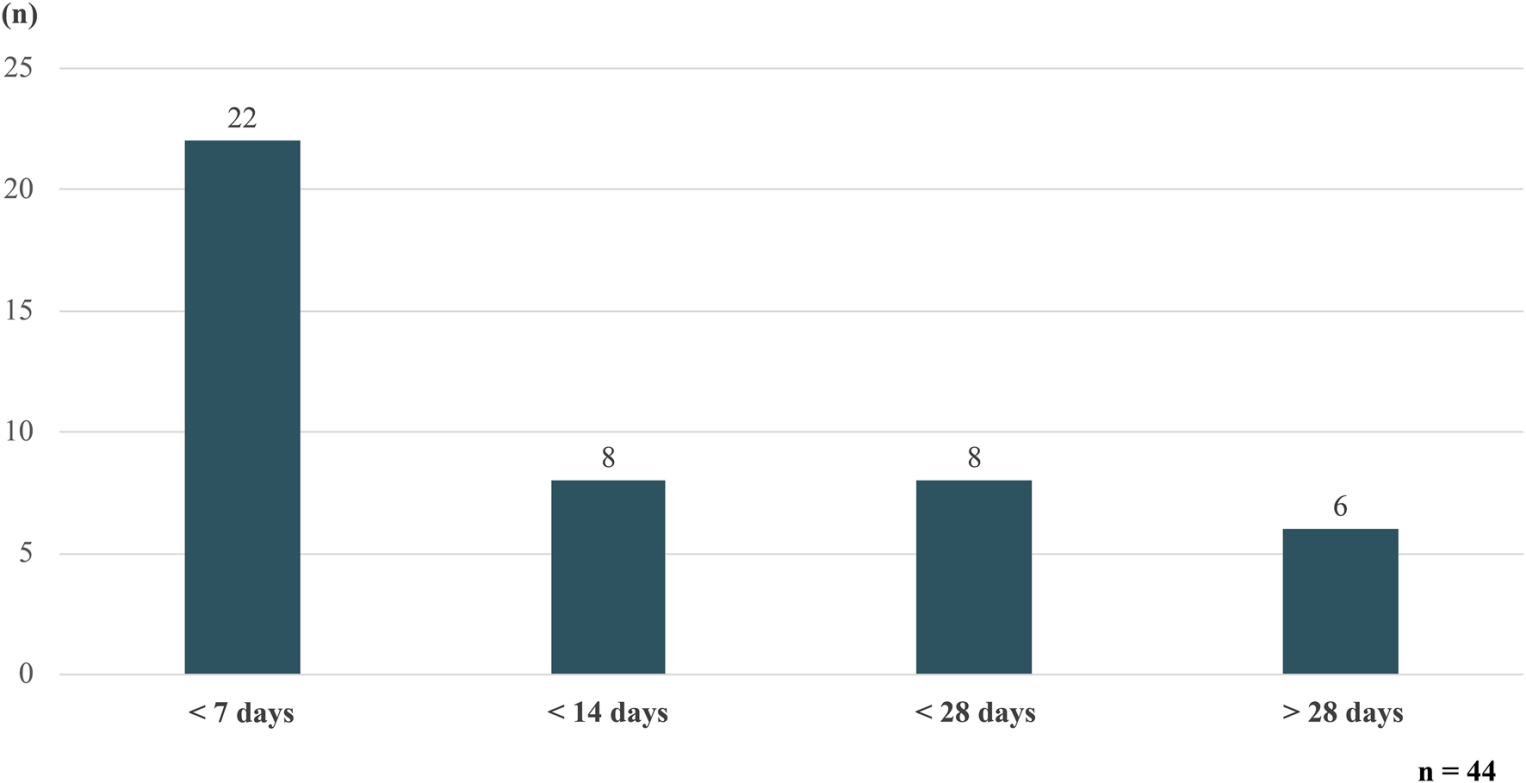
Interval between the last psychiatric visit and suicide.

This case illustrates that suicide risk may increase rapidly as childbirth and parenting become imminent, even among women receiving appropriate psychiatric follow‐up. Analysis of the interval between the last psychiatric consultation and suicide (Figure 6) demonstrates that the largest proportion of suicides occurred within 7 days of the final psychiatric visit.

The reported cases included women who were advised to undergo hospitalization but were managed as outpatients because they or their families did not consent, women whose initial psychiatric consultation was delayed despite signs of worsening mental status, and women who appeared stable during psychiatric evaluation, making suicide risk difficult to predict. In addition, some women had no prior psychiatric history but experienced a sudden deterioration in mental health. These findings suggest that psychiatric consultation alone does not necessarily prevent suicide and underscore the importance of careful follow‐up after assessment.

Prevention of maternal suicide requires structured regional collaboration among obstetric services, psychiatric care providers, and administrative agencies. Obstetricians and midwives, who maintain regular contact with pregnant and postpartum women, are in a key position to recognize subtle psychological changes and should actively assess mental health in addition to physical status. Clear regional protocols are needed to ensure timely access to appropriate psychiatric care, including hospitalization when acute risk is identified. Administrative agencies, such as public health centers and maternal–child health services, play an essential role in providing home visits, postpartum support programs, and continuous monitoring after discharge.

**FIGURE 7:**
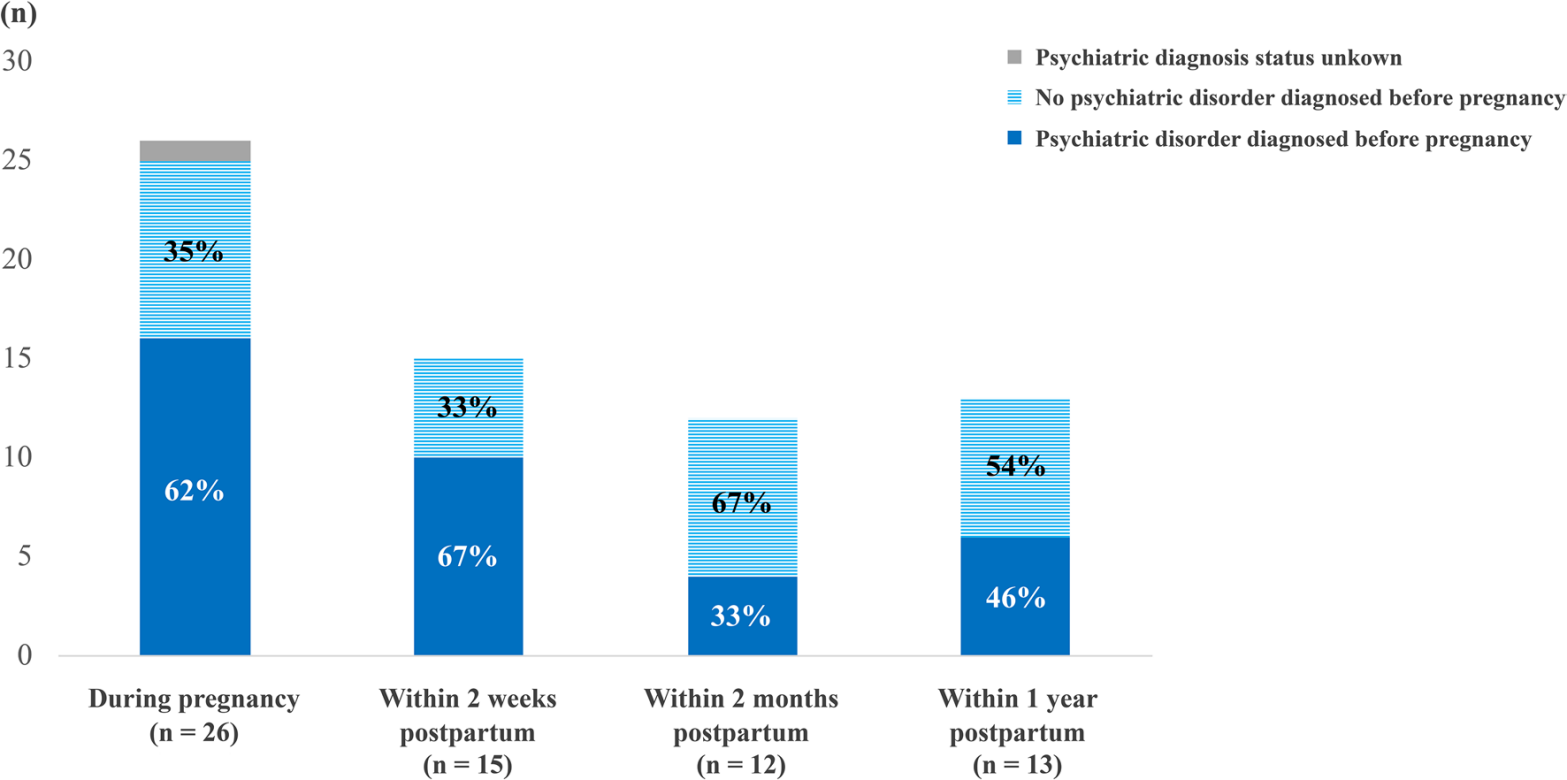
Pre‐pregnancy psychiatric diagnosis status by timing of suicide.

Importantly, Figure 7 shows that a substantial proportion of maternal suicides occurred in women without a pre‐existing psychiatric diagnosis. This finding indicates that suicide risk is not limited to traditionally defined high‐risk groups. Therefore, a population‐based approach to perinatal mental health care—providing universal screening, routine psychosocial assessment, and normalization of mental health discussions within obstetric care—is strongly recommended to reduce maternal suicide.

## 6 Proposal 5

Appropriate and proactive intervention for hyperemesis gravidarum, recognizing its association with severe physical complications and maternal mental health deterioration.

### 6.1 Case Summary 1

A multiparous woman in her 30s with persistent nausea and vomiting was instructed to remain on strict bed rest at home for threatened miscarriage. Despite severe dehydration and minimal oral intake, she did not seek reassessment. Two weeks later she was found collapsed at home and died from massive pulmonary thromboembolism, likely triggered by dehydration and prolonged immobility associated with hyperemesis gravidarum.

### 6.2 Case Summary 2

A primiparous woman in her 40s developed severe hyperemesis gravidarum after in vitro fertilization and required prolonged hospitalization. She expressed significant psychological distress and regret about the pregnancy. Although her physical condition improved and she delivered by cesarean section at term, she struggled with childcare after discharge and died by suicide 1 month postpartum, highlighting the need for careful mental health assessment and continued support.

### 6.3 Comment

Nausea, vomiting, and appetite loss are common in early pregnancy, but hyperemesis gravidarum accompanied by weight loss and dehydration requires strict medical management and should never be underestimated. hyperemesis gravidarum can lead to life‐threatening complications such as electrolyte imbalance, Wernicke encephalopathy due to thiamine deficiency, thromboembolism related to dehydration and immobility, gastrointestinal bleeding, and fetal complications including vitamin K deficiency–related hemorrhage [7]. Moreover, hyperemesis gravidarum is associated with depression, anxiety, and postpartum mental health disorders [8]. Under the recognition that hyperemesis gravidarum is a serious condition requiring multidisciplinary care, clinicians should actively prevent dehydration, avoid unnecessary immobilization, provide comprehensive nutritional and vitamin support, and assess psychological well‐being, responding promptly and appropriately when concerns arise [9].

## Author Contributions

**Tomoaki Ikeda:** conceptualization, methodology, project administration, writing – original draft, writing – review and editing.

## Funding

This work was supported by Ministry of Health, Labour and Welfare Program, 24IA2005.

## Disclosure

The authors have nothing to report.

## Conflicts of Interest

The authors declare no conflicts of interest.

## Data Availability

The data that support the findings of this study are available on request from the corresponding author. The data are not publicly available due to privacy or ethical restrictions.

## Appendix A Japan Maternal Death Exploratory Committee Members

Murasaki Aman, Tatsuya Arakaki, Masayuki Endo, Junichi Hasegawa, Koji Hashii, Masako Hayashi, Yuki Hosokawa, Tomoaki Ikeda, Hiroshi Ishikawa, Isamu Ishiwata, Chizuko A Kamiya, Takeshi Kanagawa, Naohiro Kanayama, Rie Kato, Shinji Katsuragi, Takao Kobayashi, Tomomi Kotani, Takahiko Kubo, Kentaro Kurasawa, Yoshiki Maeda, Takahide Maenaka, Shintaro Makino, Hiroshi Matsumoto, Kiyonori Miura, Takeshi Murakoshi, Akihito Nakai, Masamitsu Nakamura, Masahiko Nakata, Masafumi Nii, Keiji Nogami, Tomoaki Oda, Toshiyuki Okutomi, Kazuhiro Osato, Yoko Sagara, Atsushi Sakurai, Shoji Sato, Akihiko Sekizawa, Yumi Shina, Hiroyuki Sumikura, Toshihito Suzuki, Jun C Takahashi, Mayumi Takano, Satoru Takeda, Noboru Tanabe, Kayo Tanaka, Hiroaki Tanaka, Katsuo Terui, Nobuya Unno, Tomoko Wakasa, Tomoyuki Yamashita, Kazuma Yamakawa, Takaaki Yasuda, Jun Yoshimatsu.

**Affiliations**

Miyazaki University School of Medicine, Miyazaki, Japan; Showa Medical University School of Medicine, Tokyo, Japan; Osaka University Graduate School of Medicine, Osaka, Japan; St. Marianna University School of Medicine, Kanagawa, Japan; Hashii Women's Hospital, Kyoto, Japan; Nippon Medical School Tama Nagayama Hospital, Tokyo, Japan; Showa Medical University School of Medicine, Tokyo, Japan; Saiseikai Matsusaka General Hospital, Mie, Japan; Kanagawa Children's Medical Center, Kanagawa, Japan; Ishiwata Obstetrics and Gynecology Hospital, Ibaraki, Japan; National Cerebral and Cardiovascular Center, Osaka, Japan; National Cerebral and Cardiovascular Center, Osaka, Japan; Shizuoka College of Medical Care Science, Shizuoka, Japan; Showa Medical University School of Medicine, Tokyo, Japan; Miyazaki University School of Medicine, Miyazaki, Japan; Hamamatsu Medical Center, Shizuoka, Japan; Hamamatsu University School of Medicine, Shizuoka, Japan; Shirota Obstetrics and Gynecological Hospital, Kanagawa, Japan; Yokohama Municipal Citizen's Hospital, Kanagawa, Japan; Kuwana City Medical Center, Mie, Japan; Higashiosaka City Medical Center, Osaka, Japan; Urayasu Hospital, Juntendo University, Chiba, Japan; Osaka University Graduate School of Medicine, Osaka, Japan; Nagasaki University Graduate School of Biomedical Sciences, Nagasaki, Japan; Seirei Hamamatsu General Hospital, Shizuoka, Japan; Aiiku Maternal and Child Health Center, Tokyo, Japan; Fujita Health University, Aichi, Japan; Toho University Omori Medical Center, Tokyo, Japan; Mie University School of Medicine, Mie, Japan; Nara Medical University, Nara, Japan; Hamamatsu University School of Medicine, Shizuoka, Japan; St. Marianna University Yokohama Seibu Hospital, Kanagawa, Japan; Saiseikai Matsusaka General Hospital, Mie, Japan; Showa Medical University School of Medicine, Tokyo, Japan; Nihon University School of Medicine, Tokyo, Japan; Oita Prefectural Hospital Bureau, Oita, Japan; Showa Medical University School of Medicine, Tokyo, Japan; St. Luke's International Hospital, Tokyo, Japan; Yokohama City University Medical Center, Kanagawa, Japan; Juntendo Koshigaya Hospital, Juntendo University Faculty of Medicine, Saitama, Japan; Kindai University Faculty of Medicine, Osaka, Japan; Toho University Omori Medical Center, Tokyo, Japan; Juntendo University School of Medicine, Tokyo, Japan; Nakamura Hirai Tanabe Law Office, Osaka, Japan; Matsubase Ladies Clinic, Kumamoto, Japan; Matsubase Ladies Clinic, Kumamoto, Japan; Saitama Medical Center, Saitama Medical University, Saitama, Japan; JCHO Sagamino Hospital, Kanagawa, Japan; Kindai University Nara Hospital, Nara, Japan; Japanese Red Cross Medical Center, Tokyo, Japan; Osaka Medical and Pharmaceutical University, Osaka, Japan; Saitama Medical Center, Saitama Medical University, Saitama, Japan; National Cerebral and Cardiovascular Center, Osaka, Japan
